# 8-Hy­droxy-2-methyl­quinolinium dichlorido(2-methyl­quinolin-8-olato-κ^2^ *N*,*O*)zincate acetonitrile disolvate

**DOI:** 10.1107/S1600536811032338

**Published:** 2011-08-27

**Authors:** Ezzatollah Najafi, Mostafa M. Amini, Seik Weng Ng

**Affiliations:** a Department of Chemistry, General Campus, Shahid Beheshti University, Tehran 1983963113, Iran; b Department of Chemistry, University of Malaya, 50603 Kuala Lumpur, Malaysia; c Chemistry Department, Faculty of Science, King Abdulaziz University, PO Box 80203 Jeddah, Saudi Arabia

## Abstract

The reaction of 2-methyl-8-hy­droxy­quinoline and zinc chloride in acetonitrile affords the title solvated salt, (C_10_H_10_NO)[Zn(C_10_H_8_NO)Cl_2_]·2CH_3_CN, in which the Zn^II^ atom is coordinated by an­ *N*,*O*-chelating 2-methyl­quinolin-8-olate ligand and two chloride ligands in a distorted tetra­hedral geometry. The cation is linked to the heterocyclic anion by an O—H⋯O hydrogen bond and the quinolinium H atom forms a inter­molecular N—H⋯N hydrogen bond with one of the acetonitrile solvent mol­ecules.

## Related literature

For related structures, see: Najafi *et al.* (2010*a* ▶,*b* ▶); Sattarzadeh *et al.* (2009 ▶).
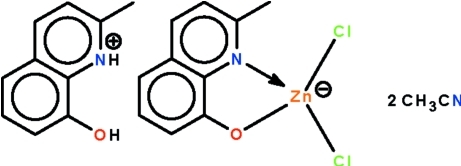


## Experimental

### 

#### Crystal data

(C_10_H_10_NO)[Zn(C_10_H_8_NO)Cl_2_]·2C_2_H_3_N
*M* *_r_* = 536.74
Monoclinic, 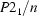

*a* = 9.9913 (2) Å
*b* = 23.1642 (5) Å
*c* = 10.4317 (2) Å
β = 95.687 (2)°
*V* = 2402.43 (8) Å^3^
*Z* = 4
Mo *K*α radiation
μ = 1.27 mm^−1^
*T* = 100 K
0.35 × 0.30 × 0.25 mm

#### Data collection

Agilent SuperNova Dual diffractometer with an Atlas detector
Absorption correction: multi-scan (*CrysAlis PRO*; Agilent, 2010 ▶) *T*_min_ = 0.664, *T*_max_ = 0.741
11981 measured reflections
5349 independent reflections
4576 reflections with *I* > 2σ(*I*)
*R*_int_ = 0.026

#### Refinement

*R*[*F* ^2^ > 2σ(*F* ^2^)] = 0.029
*wR*(*F* ^2^) = 0.070
*S* = 1.04
5349 reflections
310 parameters
2 restraints
H atoms treated by a mixture of independent and constrained refinement
Δρ_max_ = 0.38 e Å^−3^
Δρ_min_ = −0.38 e Å^−3^

### 

Data collection: *CrysAlis PRO* (Agilent, 2010 ▶); cell refinement: *CrysAlis PRO*; data reduction: *CrysAlis PRO*; program(s) used to solve structure: *SHELXS97* (Sheldrick, 2008 ▶); program(s) used to refine structure: *SHELXL97* (Sheldrick, 2008 ▶); molecular graphics: *X-SEED* (Barbour, 2001 ▶); software used to prepare material for publication: *publCIF* (Westrip, 2010 ▶).

## Supplementary Material

Crystal structure: contains datablock(s) global, I. DOI: 10.1107/S1600536811032338/lh5305sup1.cif

Structure factors: contains datablock(s) I. DOI: 10.1107/S1600536811032338/lh5305Isup2.hkl

Additional supplementary materials:  crystallographic information; 3D view; checkCIF report

## Figures and Tables

**Table 1:** Hydrogen-bond geometry (Å, °)

*D*—H⋯*A*	*D*—H	H⋯*A*	*D*⋯*A*	*D*—H⋯*A*
N2—H1⋯N3	0.87 (1)	2.15 (1)	2.988 (2)	161 (2)
O2—H2⋯O1	0.84 (1)	1.71 (1)	2.554 (2)	176 (3)

**Fig. 1.**
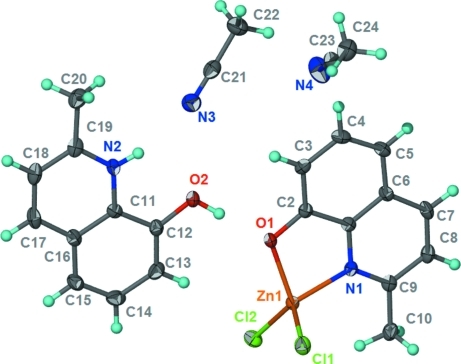
Thermal ellipsoid plot (Barbour, 2001) of (C10H10NO)[ZnCl2(C10H8NO)].2CH3CN at the 70% probability level; hydrogen atoms are drawn as spheres of arbitrary radius.

## supplementary crystallographic information

### Comment

We have synthesized methanol solvated 8-hydroxy-2-methylquinolinium dihalo(2-methylquinolin-8-olato)zincates(II) by the direct reaction of a zinc halide and 8-hydroxy-2-methylquinoline in methanol. In these salts, the Zn^II^ ion is in a tetrahedral geometry, and the ion-pairs are linked to the solvent molecules by hydrogen bonds (Najafi *et al.*, 2010*a*; Najafi *et al.*, 2010*b*; Sattarzadeh *et al.*, 2009). In the present study, the corresponding reaction of zinc chloride and the quinoline in acetonitrile yielded an analogous solvated salt (Fig. 1). In (C_10_H_10_NO)[ZnCl_2_(C_10_H_8_NO)]^.^2CH_3_CN, the metal in the anion is *N*,*O*-chelated by the deprotonated ligand and it exists in a distorted tetrahedral geometry. The cation is linked to the anion by an O–H···O hydrogen bond and the quinolinium H atom forms a hydrogen bond with one of the solvent molecules (Table 1).

### Experimental

Zinc chloride (0.10 g, 0.75 mmol) and 2-methyl-8-hydroxyquinoline (0.24 g, 1.5 mmol) were loaded into a convection tube and the tube was filled with acetonitrile and kept at 333 K. Yellow crystals were collected from the side arm after several days.

### Refinement

Carbon-bound H-atoms were placed in calculated positions [C—H 0.95 to 0.98 Å, *U*_iso_(H) 1.2 to 1.5*U*_eq_(C)] and were included in the refinement in the riding model approximation. The N and O bound H atoms were located in a difference Fourier map, and were refined with distance restraints of N–H 0.88±0.01, O–H 0.84±0.01 Å; their U_iso_(H) parameters were refined. The (5 6 11) reflection was removed.

### Crystal data

**Table:**

(C_10_H_10_NO)[Zn(C_10_H_8_NO)Cl_2_]·2C_2_H_3_N	*F*(000) = 1104
*M**_r_* = 536.74	*D*_x_ = 1.484 Mg m^−^^3^
Monoclinic, *P*2_1_/*n*	Mo *K*α radiation, λ = 0.71073 Å
Hall symbol: -P 2yn	Cell parameters from 6293 reflections
*a* = 9.9913 (2) Å	θ = 2.6–27.5°
*b* = 23.1642 (5) Å	µ = 1.27 mm^−^^1^
*c* = 10.4317 (2) Å	*T* = 100 K
β = 95.687 (2)°	Block, yellow
*V* = 2402.43 (8) Å^3^	0.35 × 0.30 × 0.25 mm
*Z* = 4	

### Data collection

**Table:**

Agilent SuperNova Dual diffractometer with an Atlas detector	5349 independent reflections
Radiation source: SuperNova (Mo) X-ray Source	4576 reflections with *I* > 2σ(*I*)
Mirror	*R*_int_ = 0.026
Detector resolution: 10.4041 pixels mm^-1^	θ_max_ = 27.6°, θ_min_ = 2.6°
ω scans	*h* = −10→13
Absorption correction: multi-scan (*CrysAlis PRO*; Agilent, 2010)	*k* = −19→30
*T*_min_ = 0.664, *T*_max_ = 0.741	*l* = −13→12
11981 measured reflections	

### Refinement

**Table:**

Refinement on *F*^2^	Primary atom site location: structure-invariant direct methods
Least-squares matrix: full	Secondary atom site location: difference Fourier map
*R*[*F*^2^ > 2σ(*F*^2^)] = 0.029	Hydrogen site location: inferred from neighbouring sites
*wR*(*F*^2^) = 0.070	H atoms treated by a mixture of independent and constrained refinement
*S* = 1.04	*w* = 1/[σ^2^(*F*_o_^2^) + (0.0274*P*)^2^ + 0.9529*P*] where *P* = (*F*_o_^2^ + 2*F*_c_^2^)/3
5349 reflections	(Δ/σ)_max_ = 0.001
310 parameters	Δρ_max_ = 0.38 e Å^−^^3^
2 restraints	Δρ_min_ = −0.38 e Å^−^^3^

### Fractional atomic coordinates and isotropic or equivalent isotropic displacement parameters (Å^2^)

**Table:**

	*x*	*y*	*z*	*U*_iso_*/*U*_eq_	
Zn1	0.50594 (2)	0.616546 (9)	0.82369 (2)	0.01365 (7)	
Cl1	0.29181 (4)	0.60211 (2)	0.86105 (4)	0.02035 (11)	
Cl2	0.59332 (5)	0.54481 (2)	0.71722 (5)	0.02002 (11)	
O1	0.52662 (12)	0.69544 (5)	0.75329 (12)	0.0166 (3)	
O2	0.52356 (13)	0.74218 (6)	0.53126 (12)	0.0183 (3)	
N1	0.62045 (14)	0.64878 (7)	0.98072 (14)	0.0129 (3)	
N2	0.43930 (15)	0.79211 (7)	0.30728 (15)	0.0147 (3)	
N3	0.48880 (18)	0.88039 (8)	0.51544 (17)	0.0257 (4)	
N4	0.4352 (2)	0.95235 (9)	0.86812 (19)	0.0363 (5)	
C1	0.62800 (17)	0.70784 (8)	0.96775 (17)	0.0134 (4)	
C2	0.57490 (17)	0.73143 (8)	0.84652 (17)	0.0137 (4)	
C3	0.57620 (17)	0.79080 (8)	0.83285 (18)	0.0160 (4)	
H3	0.5408	0.8078	0.7538	0.019*	
C4	0.62944 (18)	0.82644 (8)	0.93482 (19)	0.0186 (4)	
H4	0.6277	0.8671	0.9234	0.022*	
C5	0.68383 (17)	0.80403 (8)	1.05027 (18)	0.0169 (4)	
H5	0.7204	0.8289	1.1173	0.020*	
C6	0.68473 (17)	0.74367 (8)	1.06793 (17)	0.0145 (4)	
C7	0.73945 (17)	0.71553 (9)	1.18220 (18)	0.0176 (4)	
H7	0.7802	0.7376	1.2522	0.021*	
C8	0.73379 (18)	0.65707 (9)	1.19184 (18)	0.0178 (4)	
H8	0.7720	0.6385	1.2680	0.021*	
C9	0.67119 (17)	0.62375 (8)	1.08874 (18)	0.0151 (4)	
C10	0.6608 (2)	0.55949 (8)	1.09901 (18)	0.0201 (4)	
H10A	0.6233	0.5436	1.0161	0.030*	
H10B	0.6019	0.5496	1.1655	0.030*	
H10C	0.7504	0.5432	1.1222	0.030*	
C11	0.43571 (17)	0.73305 (8)	0.31627 (17)	0.0143 (4)	
C12	0.48188 (17)	0.70639 (8)	0.43413 (17)	0.0151 (4)	
C13	0.48303 (19)	0.64703 (8)	0.43976 (18)	0.0194 (4)	
H13	0.5167	0.6281	0.5171	0.023*	
C14	0.4344 (2)	0.61412 (9)	0.33107 (19)	0.0219 (4)	
H14	0.4348	0.5732	0.3370	0.026*	
C15	0.38685 (19)	0.63977 (9)	0.21745 (19)	0.0206 (4)	
H15	0.3539	0.6168	0.1456	0.025*	
C16	0.38707 (17)	0.70040 (8)	0.20771 (17)	0.0165 (4)	
C17	0.34114 (18)	0.73119 (9)	0.09457 (18)	0.0198 (4)	
H17	0.3041	0.7107	0.0205	0.024*	
C18	0.34947 (18)	0.78995 (9)	0.09070 (18)	0.0203 (4)	
H18	0.3197	0.8099	0.0135	0.024*	
C19	0.40153 (18)	0.82124 (9)	0.19951 (18)	0.0180 (4)	
C20	0.4180 (2)	0.88510 (9)	0.1995 (2)	0.0242 (4)	
H20A	0.4989	0.8957	0.2554	0.036*	
H20B	0.3392	0.9032	0.2316	0.036*	
H20C	0.4267	0.8984	0.1116	0.036*	
C21	0.54598 (19)	0.91734 (9)	0.56751 (19)	0.0209 (4)	
C22	0.6214 (2)	0.96503 (9)	0.6311 (2)	0.0286 (5)	
H22A	0.6586	0.9530	0.7174	0.043*	
H22B	0.5614	0.9981	0.6381	0.043*	
H22C	0.6949	0.9761	0.5804	0.043*	
C23	0.3541 (2)	0.98177 (9)	0.90117 (19)	0.0235 (4)	
C24	0.2502 (2)	1.01887 (10)	0.9443 (2)	0.0283 (5)	
H24A	0.1619	1.0059	0.9057	0.042*	
H24B	0.2656	1.0587	0.9180	0.042*	
H24C	0.2531	1.0170	1.0384	0.042*	
H1	0.463 (2)	0.8111 (10)	0.3782 (15)	0.041 (7)*	
H2	0.528 (3)	0.7256 (11)	0.6035 (15)	0.050 (8)*	

### Atomic displacement parameters (Å^2^)

**Table:**

	*U*^11^	*U*^22^	*U*^33^	*U*^12^	*U*^13^	*U*^23^
Zn1	0.01575 (11)	0.01283 (11)	0.01221 (11)	−0.00044 (8)	0.00062 (8)	−0.00073 (8)
Cl1	0.0171 (2)	0.0268 (3)	0.0173 (2)	−0.00230 (19)	0.00304 (18)	−0.0017 (2)
Cl2	0.0212 (2)	0.0164 (2)	0.0227 (2)	0.00204 (18)	0.00295 (19)	−0.00457 (19)
O1	0.0234 (7)	0.0148 (7)	0.0112 (6)	−0.0032 (5)	−0.0013 (5)	−0.0007 (5)
O2	0.0248 (7)	0.0178 (7)	0.0116 (7)	−0.0021 (6)	−0.0013 (6)	0.0010 (6)
N1	0.0124 (7)	0.0145 (8)	0.0118 (7)	0.0016 (6)	0.0011 (6)	0.0007 (6)
N2	0.0151 (7)	0.0165 (8)	0.0125 (8)	0.0017 (6)	0.0013 (6)	−0.0015 (7)
N3	0.0297 (9)	0.0237 (10)	0.0245 (9)	−0.0034 (8)	0.0065 (8)	−0.0017 (8)
N4	0.0380 (11)	0.0362 (12)	0.0327 (11)	0.0087 (9)	−0.0070 (9)	−0.0074 (9)
C1	0.0113 (8)	0.0146 (9)	0.0147 (9)	0.0002 (7)	0.0036 (7)	−0.0016 (7)
C2	0.0127 (8)	0.0152 (9)	0.0136 (9)	0.0003 (7)	0.0030 (7)	−0.0001 (7)
C3	0.0148 (8)	0.0162 (9)	0.0170 (9)	−0.0001 (7)	0.0016 (7)	0.0033 (8)
C4	0.0178 (9)	0.0131 (9)	0.0252 (10)	−0.0010 (7)	0.0042 (8)	−0.0019 (8)
C5	0.0140 (8)	0.0166 (9)	0.0199 (10)	−0.0025 (7)	0.0006 (8)	−0.0046 (8)
C6	0.0102 (8)	0.0181 (9)	0.0153 (9)	−0.0020 (7)	0.0020 (7)	−0.0031 (8)
C7	0.0142 (8)	0.0238 (10)	0.0143 (9)	−0.0012 (8)	−0.0009 (7)	−0.0049 (8)
C8	0.0165 (9)	0.0231 (10)	0.0132 (9)	0.0029 (8)	−0.0015 (7)	0.0022 (8)
C9	0.0126 (8)	0.0169 (9)	0.0158 (9)	0.0021 (7)	0.0020 (7)	0.0001 (8)
C10	0.0249 (10)	0.0192 (10)	0.0158 (9)	0.0025 (8)	−0.0005 (8)	0.0033 (8)
C11	0.0119 (8)	0.0154 (9)	0.0162 (9)	−0.0007 (7)	0.0043 (7)	−0.0016 (8)
C12	0.0137 (8)	0.0189 (10)	0.0132 (9)	−0.0001 (7)	0.0035 (7)	−0.0016 (8)
C13	0.0226 (9)	0.0195 (10)	0.0171 (9)	0.0019 (8)	0.0075 (8)	0.0027 (8)
C14	0.0262 (10)	0.0159 (10)	0.0256 (11)	−0.0026 (8)	0.0121 (9)	−0.0042 (8)
C15	0.0218 (10)	0.0217 (10)	0.0194 (10)	−0.0056 (8)	0.0076 (8)	−0.0079 (9)
C16	0.0129 (8)	0.0219 (10)	0.0153 (9)	−0.0020 (7)	0.0047 (7)	−0.0039 (8)
C17	0.0136 (9)	0.0319 (12)	0.0140 (9)	0.0006 (8)	0.0016 (7)	−0.0058 (8)
C18	0.0168 (9)	0.0300 (11)	0.0140 (9)	0.0058 (8)	0.0010 (8)	0.0025 (8)
C19	0.0141 (8)	0.0235 (10)	0.0165 (9)	0.0055 (8)	0.0027 (7)	0.0017 (8)
C20	0.0289 (11)	0.0211 (11)	0.0227 (11)	0.0063 (9)	0.0034 (9)	0.0043 (9)
C21	0.0220 (10)	0.0218 (11)	0.0198 (10)	0.0029 (9)	0.0067 (8)	0.0030 (9)
C22	0.0331 (11)	0.0223 (11)	0.0300 (11)	−0.0030 (9)	0.0008 (10)	−0.0029 (9)
C23	0.0268 (10)	0.0249 (11)	0.0169 (10)	−0.0051 (9)	−0.0066 (8)	0.0021 (9)
C24	0.0300 (11)	0.0302 (12)	0.0249 (11)	0.0025 (9)	0.0034 (9)	0.0028 (10)

### Geometric parameters (Å, °)

**Table:**

Zn1—O1	1.9880 (13)	C10—H10A	0.9800
Zn1—N1	2.0441 (15)	C10—H10B	0.9800
Zn1—Cl2	2.2246 (5)	C10—H10C	0.9800
Zn1—Cl1	2.2375 (5)	C11—C16	1.408 (3)
O1—C2	1.334 (2)	C11—C12	1.412 (3)
O2—C12	1.343 (2)	C12—C13	1.376 (3)
O2—H2	0.843 (10)	C13—C14	1.412 (3)
N1—C9	1.323 (2)	C13—H13	0.9500
N1—C1	1.378 (2)	C14—C15	1.368 (3)
N2—C19	1.333 (2)	C14—H14	0.9500
N2—C11	1.372 (2)	C15—C16	1.408 (3)
N2—H1	0.874 (10)	C15—H15	0.9500
N3—C21	1.137 (3)	C16—C17	1.416 (3)
N4—C23	1.138 (3)	C17—C18	1.365 (3)
C1—C6	1.409 (2)	C17—H17	0.9500
C1—C2	1.431 (2)	C18—C19	1.403 (3)
C2—C3	1.383 (3)	C18—H18	0.9500
C3—C4	1.408 (3)	C19—C20	1.488 (3)
C3—H3	0.9500	C20—H20A	0.9800
C4—C5	1.373 (3)	C20—H20B	0.9800
C4—H4	0.9500	C20—H20C	0.9800
C5—C6	1.410 (3)	C21—C22	1.459 (3)
C5—H5	0.9500	C22—H22A	0.9800
C6—C7	1.420 (3)	C22—H22B	0.9800
C7—C8	1.360 (3)	C22—H22C	0.9800
C7—H7	0.9500	C23—C24	1.453 (3)
C8—C9	1.418 (3)	C24—H24A	0.9800
C8—H8	0.9500	C24—H24B	0.9800
C9—C10	1.497 (3)	C24—H24C	0.9800
			
O1—Zn1—N1	83.67 (6)	N2—C11—C16	119.35 (17)
O1—Zn1—Cl2	116.23 (4)	N2—C11—C12	119.16 (16)
N1—Zn1—Cl2	117.18 (4)	C16—C11—C12	121.50 (17)
O1—Zn1—Cl1	109.75 (4)	O2—C12—C13	125.73 (17)
N1—Zn1—Cl1	112.58 (4)	O2—C12—C11	115.91 (16)
Cl2—Zn1—Cl1	113.870 (19)	C13—C12—C11	118.34 (17)
C2—O1—Zn1	110.39 (11)	C12—C13—C14	120.30 (18)
C12—O2—H2	112.2 (19)	C12—C13—H13	119.9
C9—N1—C1	119.93 (16)	C14—C13—H13	119.9
C9—N1—Zn1	131.25 (13)	C15—C14—C13	121.57 (18)
C1—N1—Zn1	108.42 (11)	C15—C14—H14	119.2
C19—N2—C11	123.72 (17)	C13—C14—H14	119.2
C19—N2—H1	119.3 (17)	C14—C15—C16	119.51 (18)
C11—N2—H1	116.9 (17)	C14—C15—H15	120.2
N1—C1—C6	122.28 (16)	C16—C15—H15	120.2
N1—C1—C2	116.55 (16)	C11—C16—C15	118.74 (17)
C6—C1—C2	121.17 (17)	C11—C16—C17	117.22 (17)
O1—C2—C3	123.55 (17)	C15—C16—C17	124.04 (18)
O1—C2—C1	118.77 (16)	C18—C17—C16	120.76 (18)
C3—C2—C1	117.68 (17)	C18—C17—H17	119.6
C2—C3—C4	120.78 (17)	C16—C17—H17	119.6
C2—C3—H3	119.6	C17—C18—C19	120.69 (18)
C4—C3—H3	119.6	C17—C18—H18	119.7
C5—C4—C3	121.86 (18)	C19—C18—H18	119.7
C5—C4—H4	119.1	N2—C19—C18	118.17 (18)
C3—C4—H4	119.1	N2—C19—C20	118.75 (17)
C4—C5—C6	119.09 (17)	C18—C19—C20	123.07 (18)
C4—C5—H5	120.5	C19—C20—H20A	109.5
C6—C5—H5	120.5	C19—C20—H20B	109.5
C1—C6—C5	119.36 (17)	H20A—C20—H20B	109.5
C1—C6—C7	116.46 (17)	C19—C20—H20C	109.5
C5—C6—C7	124.18 (17)	H20A—C20—H20C	109.5
C8—C7—C6	120.27 (17)	H20B—C20—H20C	109.5
C8—C7—H7	119.9	N3—C21—C22	178.3 (2)
C6—C7—H7	119.9	C21—C22—H22A	109.5
C7—C8—C9	120.30 (18)	C21—C22—H22B	109.5
C7—C8—H8	119.8	H22A—C22—H22B	109.5
C9—C8—H8	119.8	C21—C22—H22C	109.5
N1—C9—C8	120.70 (17)	H22A—C22—H22C	109.5
N1—C9—C10	118.26 (16)	H22B—C22—H22C	109.5
C8—C9—C10	121.04 (17)	N4—C23—C24	179.4 (3)
C9—C10—H10A	109.5	C23—C24—H24A	109.5
C9—C10—H10B	109.5	C23—C24—H24B	109.5
H10A—C10—H10B	109.5	H24A—C24—H24B	109.5
C9—C10—H10C	109.5	C23—C24—H24C	109.5
H10A—C10—H10C	109.5	H24A—C24—H24C	109.5
H10B—C10—H10C	109.5	H24B—C24—H24C	109.5
			
N1—Zn1—O1—C2	−13.18 (11)	C6—C7—C8—C9	−1.1 (3)
Cl2—Zn1—O1—C2	−130.42 (10)	C1—N1—C9—C8	−0.1 (2)
Cl1—Zn1—O1—C2	98.57 (10)	Zn1—N1—C9—C8	−171.82 (12)
O1—Zn1—N1—C9	−175.78 (16)	C1—N1—C9—C10	179.93 (15)
Cl2—Zn1—N1—C9	−59.49 (16)	Zn1—N1—C9—C10	8.2 (2)
Cl1—Zn1—N1—C9	75.43 (15)	C7—C8—C9—N1	1.7 (3)
O1—Zn1—N1—C1	11.78 (11)	C7—C8—C9—C10	−178.36 (17)
Cl2—Zn1—N1—C1	128.07 (10)	C19—N2—C11—C16	−1.4 (3)
Cl1—Zn1—N1—C1	−97.01 (10)	C19—N2—C11—C12	178.10 (16)
C9—N1—C1—C6	−2.1 (2)	N2—C11—C12—O2	1.6 (2)
Zn1—N1—C1—C6	171.36 (13)	C16—C11—C12—O2	−178.84 (15)
C9—N1—C1—C2	177.96 (15)	N2—C11—C12—C13	−177.21 (16)
Zn1—N1—C1—C2	−8.60 (17)	C16—C11—C12—C13	2.3 (3)
Zn1—O1—C2—C3	−167.34 (14)	O2—C12—C13—C14	179.05 (16)
Zn1—O1—C2—C1	12.42 (18)	C11—C12—C13—C14	−2.2 (3)
N1—C1—C2—O1	−2.4 (2)	C12—C13—C14—C15	0.9 (3)
C6—C1—C2—O1	177.65 (15)	C13—C14—C15—C16	0.5 (3)
N1—C1—C2—C3	177.38 (15)	N2—C11—C16—C15	178.55 (16)
C6—C1—C2—C3	−2.6 (2)	C12—C11—C16—C15	−1.0 (2)
O1—C2—C3—C4	−179.59 (16)	N2—C11—C16—C17	−1.4 (2)
C1—C2—C3—C4	0.6 (2)	C12—C11—C16—C17	179.06 (15)
C2—C3—C4—C5	1.1 (3)	C14—C15—C16—C11	−0.4 (3)
C3—C4—C5—C6	−0.9 (3)	C14—C15—C16—C17	179.50 (17)
N1—C1—C6—C5	−177.17 (16)	C11—C16—C17—C18	2.6 (2)
C2—C1—C6—C5	2.8 (2)	C15—C16—C17—C18	−177.32 (17)
N1—C1—C6—C7	2.6 (2)	C16—C17—C18—C19	−1.1 (3)
C2—C1—C6—C7	−177.46 (15)	C11—N2—C19—C18	3.0 (2)
C4—C5—C6—C1	−1.0 (3)	C11—N2—C19—C20	−176.26 (16)
C4—C5—C6—C7	179.25 (16)	C17—C18—C19—N2	−1.7 (3)
C1—C6—C7—C8	−1.0 (2)	C17—C18—C19—C20	177.56 (17)
C5—C6—C7—C8	178.77 (17)		

### Hydrogen-bond geometry (Å, °)

**Table:**

*D*—H···*A*	*D*—H	H···*A*	*D*···*A*	*D*—H···*A*
N2—H1···N3	0.87 (1)	2.15 (1)	2.988 (2)	161 (2)
O2—H2···O1	0.84 (1)	1.71 (1)	2.554 (2)	176 (3)
